# NetPath: a public resource of curated signal transduction pathways

**DOI:** 10.1186/gb-2010-11-1-r3

**Published:** 2010-01-12

**Authors:** Kumaran Kandasamy, S Sujatha Mohan, Rajesh Raju, Shivakumar Keerthikumar, Ghantasala S Sameer Kumar, Abhilash K Venugopal, Deepthi Telikicherla, J Daniel Navarro, Suresh Mathivanan, Christian Pecquet, Sashi Kanth Gollapudi, Sudhir Gopal Tattikota, Shyam Mohan, Hariprasad Padhukasahasram, Yashwanth Subbannayya, Renu Goel, Harrys KC Jacob, Jun Zhong, Raja Sekhar, Vishalakshi Nanjappa, Lavanya Balakrishnan, Roopashree Subbaiah, YL Ramachandra, B Abdul Rahiman, TS Keshava Prasad, Jian-Xin Lin, Jon CD Houtman, Stephen Desiderio, Jean-Christophe Renauld, Stefan N Constantinescu, Osamu Ohara, Toshio Hirano, Masato Kubo, Sujay Singh, Purvesh Khatri, Sorin Draghici, Gary D Bader, Chris Sander, Warren J Leonard, Akhilesh Pandey

**Affiliations:** 1 Institute of Bioinformatics, International Tech Park, Bangalore 560066, India; 2 McKusick-Nathans Institute of Genetic Medicine and the Department of Biological Chemistry, Johns Hopkins University, Baltimore, Maryland 21205, USA; 3 Current address: Research Unit for Immunoinformatics, RIKEN Research Center for Allergy and Immunology, RIKEN Yokohama Institute, Kanagawa 230-0045, Japan; 4 Department of Biotechnology and Bioinformatics, Kuvempu University, Jnanasahyadri, Shimoga 577451, India; 5 Laboratory of Molecular Immunology, National Heart, Lung, and Blood Institute, NIH, Bethesda, MD 20892, USA; 6 Department of Microbiology, Carver College of Medicine, University of Iowa, Iowa City, Iowa 52242, USA; 7 Department of Molecular Biology and Genetics, Institute for Cell Engineering, Johns Hopkins University School of Medicine, Baltimore, MD 21205, USA; 8 The Ludwig Institute for Cancer Research, Brussels Branch, and the Experimental Medicine Unit, Christian de Duve Institute of Cellular Pathology, Universite Catholique de Louvain, avenue Hippocrate 74, B-1200-Brussels, Belgium; 9 Laboratory for Immunogenomics, RIKEN Research Center for Allergy and Immunology, RIKEN Yokohama Institute, Kanagawa 230-0045, Japan; 10 Department of Human Genome Technology, Kazusa DNA Research Institute, 2-6-7 Kazusa-Kamatari, Kisarazu, Chiba 292-0818, Japan; 11 Laboratory for Cytokine Signaling, RIKEN Research Center for Allergy and Immunology, Yokohama, Kanagawa 230-0045, Japan; 12 Laboratories of Developmental Immunology, Graduate School of Frontier Biosciences and Graduate School of Medicine, Osaka University, Osaka 565-0871, Japan; 13 Research Institute for Biological Sciences, Tokyo University of Science, Yamazaki, Noda City, Chiba 278-0022, Japan; 14 Signal/Network Team, RIKEN Research Center for Allergy and Immunology, RIKEN Yokohama Institute, Suehiro-cho, Tsurumi, Yokohama, Kanagawa 230-0045, Japan; 15 IMGENEX India Pvt. Ltd., Bhubaneswar, Orissa 92121, India; 16 Department of Computer Science, Wayne State University, Detroit, Michigan 48202, USA; 17 Karmanos Cancer Institute, Wayne State University, Detroit, Michigan 48202, USA; 18 Banting and Best Department of Medical Research, Terrence Donnelly Centre for Cellular and Biomolecular Research, University of Toronto, 160 College St, Toronto, Ontario M5S 3E1, Canada; 19 Computational Biology Center, Memorial Sloan-Kettering Cancer Center, New York, New York 10021, USA; 20 Department of Oncology, Johns Hopkins University, Baltimore, Maryland 21205, USA

## Abstract

We have developed NetPath as a resource of curated human signaling pathways. As an initial step, NetPath provides detailed maps of a number of immune signaling pathways, which include approximately 1,600 reactions annotated from the literature and more than 2,800 instances of transcriptionally regulated genes - all linked to over 5,500 published articles. We anticipate NetPath to become a consolidated resource for human signaling pathways that should enable systems biology approaches.

## Abstract 2

NetPath, a novel community resource of curated human signaling pathways is presented and its utility demonstrated using immune signaling data.

## Background

Complex biological processes such as proliferation, migration and apoptosis are generally regulated through responses of cells to stimuli in their environment. Signal transduction pathways often involve binding of extracellular ligands to receptors, which trigger a sequence of biochemical reactions inside the cell. Generally, proteins are the effector molecules, which function as part of larger protein complexes in signaling cascades. Cellular signaling events are generally studied systematically through individual experiments that are widely scattered in the biomedical literature. Assembling these individual experiments and putting them in the context of a signaling pathway is difficult, time-consuming and cannot be automated.

The availability of detailed signal transduction pathways that can easily be understood by humans as well as be processed by computers is of great value to biologists trying to understand the working of cells, tissues and organ systems [1]. A systems-level understanding of any biological process requires, at the very least, a comprehensive map depicting the relationships among the various molecules involved [2]. For instance, these maps could be used to construct a complete network of protein-protein interactions and transcriptional events, which would help in identifying novel transcriptional and other regulatory networks [3]. These can be extended to predict how the interactions, if perturbed singly or in combination, could affect individual biological processes. Additionally, they could be used to identify possible unintended effects of a candidate therapeutic agent on any clusters in a pathway [4]. We have developed a resource called NetPath that allows biomedical scientists to visualize, process and manipulate data pertaining to signaling pathways in humans.

## Results and discussion

### Development of NetPath as a resource for signal transduction pathways

**Figure 1:**
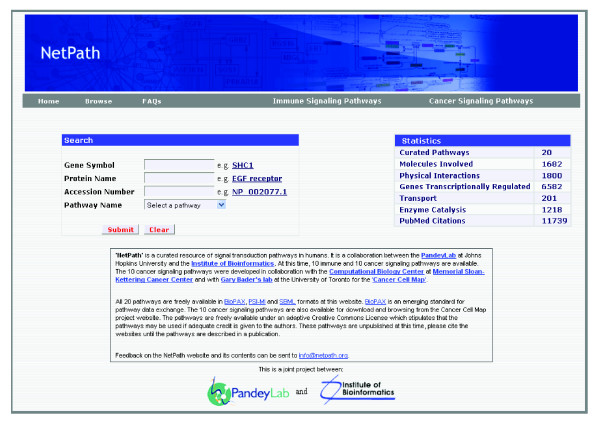
**The NetPath homepage**. The search function allows users to query the database with multiple options, including gene symbol, protein name, accession number and name of the pathway. The browse option links directly to a page listing all available pathways.

NetPath [5] is a resource for signaling pathways in humans. As an initial set, we have curated a list of ten immune signaling pathways. The list of immune signaling pathways includes T and B cell receptor signaling pathways in addition to several interleukin signaling pathways, as shown in Table 1. A query system facilitates searches based on protein/gene names or accession numbers to obtain the list of cellular signaling pathways involving the queried protein (Figure 1).

**Table 1:** Immune signaling pathway statistics

	Pathway	Molecular association events	Catalysis events	Transport events	Total reactions	Number of upregulated genes annotated	Number of downregulated genes annotated	Number of PubMed links
1	T cell receptor	202	215	13	430	453	178	1,153
2	B cell receptor	172	136	43	351	253	182	990
3	IL-1	55	44	9	108	161	79	461
4	IL-2	68	76	11	155	539	301	1289
5	IL-3	65	52	5	122	43	10	250
6	IL-4	59	47	5	111	222	90	519
7	IL-5	26	40	6	72	167	9	308
8	IL-6	65	58	7	130	84	25	332
9	IL-7	14	39	2	55	57	14	175
10	IL-9	14	20	4	38	25	1	103
Total	10	740	727	105	1,572	2,004	889	5,580

### Signaling pathway annotation

To facilitate annotation of pathway data, we first developed a tool called 'PathBuilder' [6]. PathBuilder is a signal transduction pathway annotation tool that allows annotation of pathway information, storage of data, easy retrieval and export into community standardized data structures such as BioPAX (Biological Pathways Exchange) [7], PSI-MI (Proteomics Standards Initiative - Molecular Interactions) [8] and SBML (Systems Biology Markup Language) [9] formats. PathBuilder facilitates the entry of information pertaining to protein interactions, enzyme-regulated reactions, intracellular translocation events and genes that are transcriptionally regulated.

Protein-protein interactions could be binary when two proteins directly interact with each other - 'direct interaction' - or when the proteins are present in a complex of proteins - 'complex interaction'. Both types of protein interactions are comprehensively collected from the literature. We provide PubMed identifiers, experiment type and host organism in which the interaction has been detected.

Enzyme-regulated reactions such as post-translational modifications (for example, phosphorylation, proteolytic cleavage, ubiquitination, prenylation or sulfation) are annotated as catalysis interactions. For each catalysis or modification event, the upstream enzyme, downstream targets and the site of the modification for a protein are annotated, if available. Proteins that translocate from one compartment (for example, the cytoplasm) to another (for example, the nucleus) are represented as transport events. For all reactions, a brief comment describing the reaction is also provided.

### Display of pathway information

The homepage of any given pathway contains a brief description of the pathway, a summary of the reaction statistics and a list of the molecules involved in the pathway. Reactions in a pathway are provided under three distinct categories, including physical interactions, enzyme catalysis and transport. Furthermore, the pathway data are also provided in PSI-MI, BioPAX and SBML formats, which can also be visualized through other external network visualization software, such as Cytoscape [10].

### Cataloging transcriptionally regulated genes

In addition to the above pathway annotations, information on genes that are transcriptionally regulated is provided in NetPath. This is important because addition of most extracellular growth factors or ligands leads to an alteration in the transcriptome of the cell. Often, some of the transcriptionally regulated genes are used as 'reporters' in biological experiments where the pathway is being studied. We have cataloged a number of genes that are up- or down-regulated by the particular ligand involved in each pathway. These up/down-regulated genes can be considered as 'signatures' for that particular pathway. We have incorporated both microarray and non-microarray (for example, Northern blot, quantitative RT-PCR, serial analysis of gene expression (SAGE), and so on) experiments for gene expression. In each case, the type of experiment (that is, microarray, non-microarray or both) used to obtain the data is indicated. Additionally, we have also annotated the transcription factors that are responsible for transcriptional regulation of the downstream genes where such information is available. Given the large number of transcriptionally regulated genes for each pathway, we have also developed a query system that permits users to search such genes using gene symbol or accession numbers. This feature will be valuable for shortlisting genes that are common to several pathways or specific to any given pathway.

### Pathway statistics

At present the 10 annotated immune signaling pathways comprise 703 proteins and 1,572 reactions. The reactions can be grouped into 740 molecular association events, 727 enzyme catalysis events and 105 translocation events. Our pathways provide a list of 2,004 and 889 genes that are up- or down-regulated, respectively, at the level of mRNA expression. Including 10 cancer signaling pathways that are also available through Cancer Cell Map [11], NetPath now contains 1,682 proteins and 3,219 reactions, which can be grouped into 1,800 molecular association events, 1,218 enzyme catalysis events and 201 transport events. Table 1 shows the overall immune signaling pathway statistics as of 1 November 2009.

### Comparison with other signaling databases

Although over 310 resources [12] provide some form of pathway related information, many of these currently available resources are databases for protein-protein interactions, metabolic pathways, transcription factors/gene regulatory networks, and genetic interaction networks. Some of these pathways include the Kyoto Encyclopedia of Genes and Genomes (KEGG) [13], BioCarta [14], Science's Signal Transduction Knowledge Environment (STKE) Connections Maps [15], Reactome [16], National Cancer Institute's Pathway Interaction Database (PID) [17], Pathway database from Cell Signaling Technology [18], Integrating Network Objects with Hierarchies (INOH) [19], Signaling Pathway Database (SPAD) [20], GOLD.db [21], PATIKA [22], pSTIING [23], TRMP [24], WikiPathways [25] and PANTHER [26]. However, many of these pathway resources are not primary - that is, they combine data from many other sources. Thus, we have compared NetPath with eight other signaling pathways that contain manually curated human pathway data derived from experiments. Of all these pathways that are compared, NetPath stands out for three unique features. The first is that it includes annotation of transcriptionally regulated genes. Such a catalog of transcriptionally regulated genes pertaining to a given pathway should be highly useful in exploring pathway-specific expression signatures. The second unique feature is that NetPath provides manually curated textual descriptions of each pathway reaction, which should facilitate an easier understanding of these pathways, aiding biomedical scientists to get an overview of the pathway reactions in a central repository. The third unique feature of NetPath is that these data can be searched using SPARQL - the recommended query language for the semantic web. Table 2 compares some of the salient features of NetPath with some of the other popular signaling pathway resources. In addition to the unique features, NetPath also provides a separate molecule page for every pathway component along with a brief textual description for each molecule. Overall, NetPath should be a useful pathway resource with unique features that should facilitate signaling research.

**Table 2:** Comparison of salient features of NetPath with other popular curated signaling pathway resources

Pathway resource	Query option for pathway molecules	Genes transcriptionally regulated by pathway included?	Pathways reviewed by experts?	File formats available for download	Textual description of reactions provided?	Other features or comments
NetPath [5]	Yes	Yes	Yes	BioPAX, PSI-MI, SBML, Excel, Tab-delimited	Yes	Focus on human receptor mediated signaling. Also contains separate molecule pages with brief summary of the biology of the individual molecules
BioCarta [14]	Yes	No	Yes	No download option provided	No	BioCarta provides commercial links to antibody reagents
Science's STKE [15]	No	No	Yes	SVG	No	Contains species-specific and also cell-type-specific pathways
KEGG [13]	Yes	No	No	KGML, BioPAX	No	Contains disease specific pathways
Reactome [16]	Yes	No	Yes	BioPAX, SBML, PDF, SVG, Protégé, MySQL database dump	Yes	Also contains computationally inferred pathway reactions
NCI-PID [17]	Yes	No	Yes	XML, BioPAX, SVG, JPG	No	Apart from NCI-Nature curated pathways, it also contains many pathways imported from BioCarta/Reactome
CST [18]	Yes	No	Yes (in some cases)	PDF	No	Provides pathway information along with links to protein and commercial products available for that protein
WikiPathways [25]	Yes	No	No	GPML, GenMAPP, PDF, PNG, SVG	No	Any user can register and create a new pathway and also edit existing pathways
PANTHER [26]	Yes	No	Reviewed by Curation Coordinator	SBML, SBGN, PNG	No	Allows community pathway curation and also provides links to Applied Biosystems genomic products

CST, Cell Signaling Technology; PID, Pathway Interaction Database; STKE, Signal Transduction Knowledge Environment.

### Interleukin-2 pathway as a prototype

One of the best studied immune signaling pathways is the interleukin (IL)-2 signaling pathway [27]. IL-2 is a multifunctional cytokine with pleiotropic effects on several cells of the immune system [27,28]. IL-2 was originally discovered as a T cell growth factor [29], but it was also found to have actions related to B cell proliferation [30], and the proliferation and cytolytic activity of natural killer cells [31]. IL-2 also activates lymphokine activated killer cells [32]. In contrast to its proliferative effects, IL-2 also has potent activity in a process known as activation-induced cell death [33]. More recently, IL-2 was shown to promote tolerance through its effects on regulatory T cell development [34]. IL-2 clinically has anti-cancer effects [35] as well as utility in supporting T cell numbers in HIV/AIDS [36].

There are three classes of IL-2 receptors, binding IL-2 with low, intermediate, or high-affinity [37]. The low affinity receptor (IL-2Rα alone) is not functional; signaling by IL-2 involves either the high affinity hetero-trimeric receptor containing IL-2Rα, IL-2Rβ and the common cytokine receptor gamma chain (originally named IL-2Rγ and now generally denoted as γc) or the intermediate affinity heterodimeric receptor composed of IL-2Rβ and γc [37,38]. Mutations in the *IL2RG *gene result in X-linked severe combined immunodeficiency disease [39]. IL-2 stimulation induces the activation of the Janus family tyrosine kinases JAK1 and JAK3, which associate with IL-2Rβ and γ_c_, respectively. These kinases in turn phosphorylate IL-2Rβ and induce tyrosine phosphorylation of STATs (signal transducers and activators of transcription) and various other downstream targets [40]. The downstream signaling pathway also involves mitogen-activated protein kinase and phosphoinositide 3-kinase signaling modules [41], leading to both mitogenic and anti-apoptotic signals [40-42].

**Figure 2:**
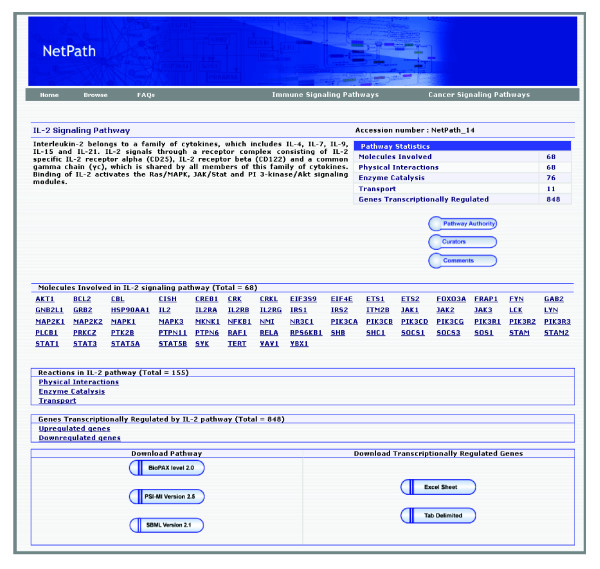
**The IL-2 pathway page in NetPath**. Hyperlinks to pathway-specific information, such as reactions, transcriptionally regulated genes, molecular associations, and catalysis events, are listed. There is also an option to download pathway information in various data exchange formats from this page.

The IL-2 signaling pathway currently comprises of 68 proteins, 155 reactions with 68 molecular association events, 76 enzymatic catalysis events and 11 translocation events. Importantly, 840 transcriptionally regulated events - that is, a list of genes up- or down-regulated by IL-2 - have been annotated from the published literature. In all, the reactions in the IL-2 pathway are supported by 1,289 links to research articles. Figure 2 shows the pathway page of the IL-2 pathway.

### Integration of pathway information with other resources

The pathways developed by us have been integrated with the Human Protein Reference Database (HPRD) [43,44]. The integration of pathways in HPRD helps identify each component of the pathway in the context of its detailed proteomic annotations [45]. As part of our community participation with other databases/resources, we hope to establish connections with other pathway databases such as KEGG [27] and Reactome [16] in the future.

### Availability of pathway data

A digital representation of pathways is essential to be able to manipulate the large amount of available information [4]. The diversity among pathway databases is also reflected in differences in data models, data access methods and file formats. This leads to the incompatibility of data formats for the analysis of pathway data. To avoid this, data standards are adopted by many of the pathway databases [12,46]. Data standards reduce the total number of translation operations needed to exchange data between multiple sources. To facilitate easy information retrieval from a wide variety of pathway resources, a broad effort in the biological pathways community called BioPAX was initiated. Since many less-detailed data types in a pathway database are difficult to represent in a very detailed format, BioPAX ontology uses hierarchical entity classes to present multiple levels of data resolution. All pathways in NetPath are available for download in BioPAX level 2, version 1.0. The PSI-MI format was developed to exchange molecular interaction data between databases containing protein-protein interactions. PSI-MI data representation facilitates data comparison, exchange and verification [8]. The molecular interaction subset of NetPath pathways is also available in PSI-MI version 2.5. SBML was developed as a medium for representation and exchange of biochemical network models [9]. NetPath provides all pathway data in SBML version 2.1 format. All data are made available under the Creative Commons license version 2.5 [47], which stipulates that the pathways may be freely used if adequate credit is given to the authors. Support for these data standards and free license enables the integration of knowledge from multiple sources in a coherent and reliable manner.

### Enabling semantic web for NetPath

The semantic web envisions an internet where specific information can be obtained from the web automatically using computers. Because providing computers with the intuitiveness of humans is nearly impossible as of now, creation of meta-data - data about data - can help computers identify what is being sought less ambiguously. However, annotating more data does not automatically imply that the data can be made easily accessible by the user. For instance, although many resources permit direct querying of individual molecules in the respective databases, queries based on 'relationships' between different entries in the databases cannot be handled. One possible solution to enable searching by such 'concepts' is to incorporate semantic web features that explicitly describe the inter-relationship between entries in the databases.

The W3C has established SPARQL as the standard semantic query language. Pathway data in BioPAX uses the web ontology language (OWL) format, which is highly descriptive in nature and can be used to make pathways semantically 'queryable'. In this regard, we have implemented an application programming interface (API) for NetPath that accepts SPARQL over HTTP to query the BioPAX files describing NetPath pathways. The return results are provided in SPARQL Query Results XML format. Although biologists cannot be expected to write SPARQL queries, the ability to send SPARQL queries over HTTP allows bioinformaticians to write client applications that can retrieve NetPath resources taking advantage of the descriptive richness of SPARQL and BioPAX.

### Analyzing impact factor for pathways

It is becoming clear that pathway information can be used in the context of genome-scale gene expression experiments. A novel approach has been recently reported to measure the biological impact of perturbation of pathways in genomewide gene expression experiments [48]. This approach considers the topology of genes in a pathway in conjunction with classical statistics for microarray analysis. The impact factor is a statistical approach that can capture the magnitude of the expression changes of each gene, the position of the differentially expressed genes on the given pathways, the topology of the pathway that describes how these genes interact, and the type of signaling interactions between them. Our previous results using KEGG pathways were found to correlate with known biological events that were missed by other widely used classical analysis methods. However, this approach could not be applied to study immune responses because of the limited availability of data on such pathways in humans.

As a proof of principle, we selected publicly available mRNA expression datasets from Gene Expression Omnibus (GEO), a repository for gene expression data [49]. Datasets that include expression analysis of immune cells under different experimental conditions were selected for this purpose.

One of the datasets used [GEO:GDS2214] (as described in [50]) was an experimental study of mRNA expression analysis of neutrophils isolated from blood of patients with sepsis-induced acute lung injury. The neutrophils were cultured with either lipopolysaccharide (LPS) or high mobility group box protein 1 (HMGB1), both of which are known to be mediators of the inflammatory response. Gene expression analysis was carried out using the Affymetrix GeneChip Human Genome U133 Array Set HG-U133A oligonucleotide gene chip. The authors found enhancement of nuclear translocation activity of NF-kappaB and phosphorylation of Akt and p38 mitogen-activated protein kinase upon stimulation of LPS or HMGB1. We carried out impact factor analysis using this dataset on all ten immune signaling pathways. The results corroborate with these findings since IL-1 and IL-6 pathway scores are highly affected while the rest of the NetPath pathways did not show significant scores.

Another dataset selected [GEO:GDS1407] (described in [51]) was a part of the gene expression study that screened a cohort of 102 healthy individuals to investigate the distribution of inflammatory responses to LPS in the normal population in circulating leukocytes. Expression profiling with Affymetrix U95AV2 oligonucleotide microarray identified differentially regulated genes between two phenotypic subgroups that have been described as high LPS responders (lps_high_) and low LPS responders (lps_low_), based on the concentration of cytokines produced in response to LPS. Gene expression analysis was done using the Affymetrix U95AV2 human oligonucleotide arrays. Impact factor analysis was carried out using this dataset on all ten immune signaling pathways. Impact factor scores for IL-1 and IL-6 NetPath pathways in the lps_high _group have high values whereas impact factor scores for lps_low _do not show any significant perturbation of NetPath pathways. The scores are consistent with experimental results showing upregulation of IL-1 and IL-6 ligands in the lps_high _group. The impact factor gives the insight that not only are the ligands upregulated, but the pathway also seems to be highly affected. It should be noted that impact factor is not the only method to measure the biological impact of perturbation of pathways and other methods will continue to be developed and could be applied to such pathway data.

### Outlook

In addition to keeping these pathways updated on a regular basis, we will also add additional pathways to NetPath. We also hope to involve the biomedical community by allowing researchers to provide feedback as well as to volunteer to become 'pathway authorities' on specific pathways, similar to the successful contribution model of the BioCarta resource [14]. In this regard, we have already recruited several investigators to serve as pathway authorities in our initial effort. Multiple pathway authorities are possible for the same pathway if there are enough interested investigators with expertise who wish to contribute in this fashion. For instance, ten other signaling pathways pertaining to cancer signaling were developed for the Cancer Cell Map project [11], as a collaboration with Memorial Sloan-Kettering Cancer Center, and these data are also available through Pathway Commons [52]. We also intend to map our human-specific pathway data to corresponding mouse orthologs to create the mouse equivalent of our signaling pathways. Since large amounts of human signaling pathway data are modeled using the mouse, this will facilitate biological system modeling that relies on primary experimental data. We also intend to incorporate pathway visualization for all existing pathways in NetPath as well as those that will be added in the future using the PathVisio software [53]. PathVisio also supports visualization of gene expression data in the context of pathways, which will enable biologists to display a systems view of the signaling pathway.

## Conclusions

We have developed a resource for integration of human cellular signaling events. These pathway-specific protein-protein interaction data can be used to generate larger physical networks of protein-protein interactions that, when coupled with data on genetic interactions, could help in defining novel functional relationships among proteins. In addition, genetic interactions can functionally link proteins that belong to unconnected physical networks. These pathways could also be used to interrogate gene expression signatures in cancers and other human diseases to better understand the mechanisms or to obtain profiles for diagnostic or therapeutic purposes. There is a large amount of known information about different cellular signaling pathways controlling a variety of cellular functions, which is difficult to collect by one group. We support the vision of many data providers collecting data of interest and making them freely available in standard formats as a scalable way to represent all known pathway information in databases for comprehensive analysis. Overall, we hope to engage the biomedical community in keeping the NetPath pathway resource up to date and as error-free as possible.

## Materials and methods

The initial annotation process of any signaling pathway involves gathering and reading of review articles to achieve a brief overview of the pathway. This process is followed by listing all the molecules that arereported to be involved in the pathway under annotation. Information regarding potential pathway authorities are also gathered at this initial stage. Pathway experts are involved in initial screening of the molecules listed to check for any obvious omissions. In the second phase, annotators manually perform extensive literature searches using search keys, which include all the alternative names of the molecules involved, the name of the pathway, the names of reactions, and so on. In addition, the iHOP [54] resource is also used to perform advanced PubMed-based literature searches to collect the reactions that were reported to be implicated in a given pathway. The collected reactions are manually entered using the PathBuilder [6] annotation interface, which is subjected to an internal review process involving PhD level scientists with expertise in the areas of molecular biology, immunology and biochemistry. However, there are instances where a molecule has been implicated in a pathway in a published report but the associated experimental evidence is either weak or differs from experiments carried out by other groups. For this purpose, we recruit several investigators as pathway authorities based on their expertise in individual signaling pathways. The review by pathway authorities occasionally leads to correction of errors or, more commonly, to inclusion of additional information. Finally, the pathway authorities help in assessing whether the work of all major laboratories has been incorporated for the given signaling pathway.

## Abbreviations

BioPAX: Biological Pathways Exchange; GEO: Gene Expression Omnibus; HMGB1: high mobility group box protein 1; HPRD: Human Protein Reference Database; IL: interleukin; KEGG: Kyoto Encyclopedia of Genes and Genomes; LPS: lipopolysaccharide; PSI-MI: Proteomics Standards Initiative - Molecular Interactions; SBML: Systems Biology Markup Language.

## Authors' contributions

SM1, RR, SK, GSSK, AKV, DT, DJN, SM2, CP, SKG, SGT, SM3, HP, YS, RG, HKCJ, JZ, RS1, VN, SB, RS2, YLR, BAR, TSKP and JL collected the data. JCDH, SD1, JR, SC, OO, TH, MK, SS, WJL and AP serve as pathway authorities. KK, SM1 and AP wrote the manuscript. KK and SM2 developed the software. KK, AKV, DJN, SKG, PK and SD carried out the impact factor analysis. KK, GDB, CS and AP participated in the study design. All authors read and approved the final manuscript.

## References

[B1] Fukuda K Takagi T Knowledge representation of signal transduction pathways. Bioinformatics 2001 17 829 837 10.1093/bioinformatics/17.9.829 11590099

[B2] Uetz P Finley RL Jr From protein networks to biological systems. FEBS Lett 2005 579 1821 1827 10.1016/j.febslet.2005.02.001 15763558

[B3] Ideker T A systems approach to discovering signaling and regulatory pathways - or, how to digest large interaction networks into relevant pieces. Adv Exp Med Biol 2004 547 21 30 15230090 10.1007/978-1-4419-8861-4_3

[B4] Schaefer CF Pathway databases. Ann N Y Acad Sci 2004 1020 77 91 10.1196/annals.1310.009 15208185

[B5] NetPath http://www.netpath.org/

[B6] Kandasamy K Keerthikumar S Raju R Keshava Prasad TS Ramachandra YL Mohan S Pandey A PathBuilder - open source software for annotating and developing pathway resources. Bioinformatics 2009 25 2860 2862 10.1093/bioinformatics/btp453 19628504 PMC2781757

[B7] BioPAX: Biological Pathways Exchange http://www.biopax.org/

[B8] Hermjakob H Montecchi-Palazzi L Bader G Wojcik J Salwinski L Ceol A Moore S Orchard S Sarkans U von Mering C Roechert B Poux S Jung E Mersch H Kersey P Lappe M Li Y Zeng R Rana D Nikolski M Husi H Brun C Shanker K Grant SG Sander C Bork P Zhu W Pandey A Brazma A Jacq B The HUPO PSI's molecular interaction format - a community standard for the representation of protein interaction data. Nat Biotechnol 2004 22 177 183 10.1038/nbt926 14755292

[B9] Hucka M Finney A Sauro HM Bolouri H Doyle JC Kitano H Arkin AP Bornstein BJ Bray D Cornish-Bowden A Cuellar AA Dronov S Gilles ED Ginkel M Gor V Goryanin II Hedley WJ Hodgman TC Hofmeyr JH Hunter PJ Juty NS Kasberger JL Kremling A Kummer U Le Novere N Loew LM Lucio D Mendes P Minch E Mjolsness ED The systems biology markup language (SBML): a medium for representation and exchange of biochemical network models. Bioinformatics 2003 19 524 531 10.1093/bioinformatics/btg015 12611808

[B10] Shannon P Markiel A Ozier O Baliga NS Wang JT Ramage D Amin N Schwikowski B Ideker T Cytoscape: a software environment for integrated models of biomolecular interaction networks. Genome Res 2003 13 2498 2504 14597658 10.1101/gr.1239303 PMC403769

[B11] The Cancer Cell Map http://cancer.cellmap.org/

[B12] Bader GD Cary MP Sander C Pathguide: a pathway resource list. Nucleic Acids Res 2006 34 D504 506 16381921 10.1093/nar/gkj126 PMC1347488

[B13] Kanehisa M Goto S KEGG: kyoto encyclopedia of genes and genomes. Nucleic Acids Res 2000 28 27 30 10592173 10.1093/nar/28.1.27 PMC102409

[B14] BioCarta http://www.biocarta.com/

[B15] Connections Maps http://stke.sciencemag.org/cm

[B16] Joshi-Tope G Gillespie M Vastrik I D'Eustachio P Schmidt E de Bono B Jassal B Gopinath GR Wu GR Matthews L Lewis S Birney E Stein L Reactome: a knowledgebase of biological pathways. Nucleic Acids Res 2005 33 D428 432 15608231 10.1093/nar/gki072 PMC540026

[B17] NCI-Nature Pathway Interaction Database http://pid.nci.nih.gov/

[B18] Cell Signaling Technology http://www.cellsignal.com/

[B19] INOH Pathway Database http://www.inoh.org/

[B20] Signaling Pathway Database http://www.grt.kyushu-u.ac.jp/spad

[B21] Hackl H Maurer M Mlecnik B Hartler J Stocker G Miranda-Saavedra D Trajanoski Z GOLD.db: genomics of lipid-associated disorders database. BMC Genomics 2004 5 93 15588328 10.1186/1471-2164-5-93 PMC544894

[B22] Demir E Babur O Dogrusoz U Gursoy A Nisanci G Cetin-Atalay R Ozturk M PATIKA: an integrated visual environment for collaborative construction and analysis of cellular pathways. Bioinformatics 2002 18 996 1003 10.1093/bioinformatics/18.7.996 12117798

[B23] Ng A Bursteinas B Gao Q Mollison E Zvelebil M pSTIING: a 'systems' approach towards integrating signalling pathways, interaction and transcriptional regulatory networks in inflammation and cancer. Nucleic Acids Res 2006 34 D527 534 16381926 10.1093/nar/gkj044 PMC1347407

[B24] Zheng CJ Zhou H Xie B Han LY Yap CW Chen YZ TRMP: a database of therapeutically relevant multiple pathways. Bioinformatics 2004 20 2236 2241 10.1093/bioinformatics/bth233 15059817

[B25] Pico AR Kelder T van Iersel MP Hanspers K Conklin BR Evelo C WikiPathways: pathway editing for the people. PLoS Biol 2008 6 e184 18651794 10.1371/journal.pbio.0060184 PMC2475545

[B26] Thomas PD Campbell MJ Kejariwal A Mi H Karlak B Daverman R Diemer K Muruganujan A Narechania A PANTHER: a library of protein families and subfamilies indexed by function. Genome Res 2003 13 2129 2141 12952881 10.1101/gr.772403 PMC403709

[B27] Nakamura M Asao H Takeshita T Sugamura K Interleukin-2 receptor heterotrimer complex and intracellular signaling. Semin Immunol 1993 5 309 317 10.1006/smim.1993.1037 8260647

[B28] Morgan DA Ruscetti FW Gallo R Selective *in vitro *growth of T lymphocytes from normal human bone marrows. Science 1976 193 1007 1008 10.1126/science.181845 181845

[B29] Mingari MC Gerosa F Carra G Accolla RS Moretta A Zubler RH Waldmann TA Moretta L Human interleukin-2 promotes proliferation of activated B cells via surface receptors similar to those of activated T cells. Nature 1984 312 641 643 10.1038/312641a0 6438535

[B30] London L Perussia B Trinchieri G Induction of proliferation *in vitro *of resting human natural killer cells: IL 2 induces into cell cycle most peripheral blood NK cells, but only a minor subset of low density T cells. J Immunol 1986 137 3845 3854 3491151

[B31] Grimm EA Mazumder A Zhang HZ Rosenberg SA Lymphokine-activated killer cell phenomenon. Lysis of natural killer-resistant fresh solid tumor cells by interleukin 2-activated autologous human peripheral blood lymphocytes. J Exp Med 1982 155 1823 1841 6176669 10.1084/jem.155.6.1823 PMC2186695

[B32] Greene WC Leonard WJ The human interleukin-2 receptor. Annu Rev Immunol 1986 4 69 95 10.1146/annurev.iy.04.040186.000441 3011033

[B33] Green DR Droin N Pinkoski M Activation-induced cell death in T cells. Immunol Rev 2003 193 70 81 10.1034/j.1600-065X.2003.00051.x 12752672

[B34] Malek TR Bayer AL Tolerance, not immunity, crucially depends on IL-2. Nat Rev Immunol 2004 4 665 674 10.1038/nri1435 15343366

[B35] Rosenberg SA Progress in human tumour immunology and immunotherapy. Nature 2001 411 380 384 10.1038/35077246 11357146

[B36] Paredes R Lopez Benaldo de Quiros JC Fernandez-Cruz E Clotet B Lane HC The potential role of interleukin-2 in patients with HIV infection. AIDS Rev 2002 4 36 40 11998783

[B37] Kim HP Imbert J Leonard WJ Both integrated and differential regulation of components of the IL-2/IL-2 receptor system. Cytokine Growth Factor Rev 2006 17 349 366 10.1016/j.cytogfr.2006.07.003 16911870

[B38] Leonard WJ Type I Cytokines and Interferons and Their Receptors. Fundamental Immunology 2008 6 Philadelphia: Lippincott Williams & Wilkins 701 749

[B39] Noguchi M Yi H Rosenblatt HM Filipovich AH Adelstein S Modi WS McBride OW Leonard WJ Interleukin-2 receptor gamma chain mutation results in X-linked severe combined immunodeficiency in humans. Cell 1993 73 147 157 10.1016/0092-8674(93)90167-O 8462096

[B40] Lin JX Leonard WJ Signaling from the IL-2 receptor to the nucleus. Cytokine Growth Factor Rev 1997 8 313 332 10.1016/S1359-6101(97)00021-X 9620644

[B41] Ellery JM Nicholls PJ Alternate signalling pathways from the interleukin-2 receptor. Cytokine Growth Factor Rev 2002 13 27 40 10.1016/S1359-6101(01)00023-5 11750878

[B42] Ahmed NN Grimes HL Bellacosa A Chan TO Tsichlis PN Transduction of interleukin-2 antiapoptotic and proliferative signals via Akt protein kinase. Proc Natl Acad Sci USA 1997 94 3627 3632 9108028 10.1073/pnas.94.8.3627 PMC20491

[B43] HPRD: Human Protein Reference Database http://www.hprd.org/

[B44] Prasad TSK Goel R Kandasamy K Keerthikumar S Kumar S Mathivanan S Telikicherla D Raju R Shafreen B Venugopal A Balakrishnan L Marimuthu A Banerjee S Somanathan DS Sebastian A Rani S Ray S Kishore CJH Kanth S Ahmed M Kashyap M Mohmood R Ramachandra YL Krishna V Rahiman AB Mohan S Ranganathan P Ramabadran S Chaerkady R Pandey A Human Protein Reference Database -- 2009 update. Nucleic Acids Res 2009 37 D767 D772 18988627 10.1093/nar/gkn892 PMC2686490

[B45] Kandasamy K Keerthikumar S Mathivanan S Patankar N Shafreen B Renuse S Pawar H Ramachandra YL Prasad TSK Acharya PK Ranganathan P Chaerkady R Pandey A Human Proteinpedia: A unified discovery resource for proteomics research. Nucleic Acids Res 2009 37 D773 D781 18948298 10.1093/nar/gkn701 PMC2686511

[B46] Cary MP Bader GD Sander C Pathway information for systems biology. FEBS Lett 2005 579 1815 1820 10.1016/j.febslet.2005.02.005 15763557

[B47] Creative Commons license version 2.5 http://creativecommons.org/licenses/by/2.5/

[B48] Draghici S Khatri P Tarca AL Amin K Done A Voichita C Georgescu C Romero R A systems biology approach for pathway level analysis. Genome Res 2007 17 1537 1545 17785539 10.1101/gr.6202607 PMC1987343

[B49] Barrett T Troup DB Wilhite SE Ledoux P Rudnev D Evangelista C Kim IF Soboleva A Tomashevsky M Edgar R NCBI GEO: mining tens of millions of expression profiles--database and tools update. Nucleic Acids Res 2007 35 D760 765 17099226 10.1093/nar/gkl887 PMC1669752

[B50] Silva E Arcaroli J He Q Svetkauskaite D Coldren C Nick JA Poch K Park JS Banerjee A Abraham E HMGB1 and LPS induce distinct patterns of gene expression and activation in neutrophils from patients with sepsis-induced acute lung injury. Intensive Care Medicine 2007 33 1829 1839 10.1007/s00134-007-0748-2 17581740

[B51] Wurfel MM Park WY Radella F Ruzinski J Sandstrom A Strout J Bumgarner RE Martin TR Identification of high and low responders to lipopolysaccharide in normal subjects: an unbiased approach to identify modulators of innate immunity. J Immunol 2005 175 2570 2578 16081831 10.4049/jimmunol.175.4.2570

[B52] Pathway Commons http://www.pathwaycommons.org/

[B53] van Iersel MP Kelder T Pico AR Hanspers K Coort S Conklin BR Evelo C Presenting and exploring biological pathways with PathVisio. BMC Bioinformatics 2008 9 399 18817533 10.1186/1471-2105-9-399 PMC2569944

[B54] Hoffmann R Valencia A A gene network for navigating the literature. Nat Genet 2004 36 664 10.1038/ng0704-664 15226743

